# Functional Avidity: A Measure to Predict the Efficacy of Effector T Cells?

**DOI:** 10.1155/2012/153863

**Published:** 2012-11-20

**Authors:** Selena Viganò, Daniel T. Utzschneider, Matthieu Perreau, Giuseppe Pantaleo, Dietmar Zehn, Alexandre Harari

**Affiliations:** ^1^Divisions of Immunology and Allergy, Department of Medicine, Centre Hospitalier Universitaire Vaudois, University of Lausanne, 1011 Lausanne, Switzerland; ^2^Swiss Vaccine Research Institute, 1011 Lausanne, Switzerland

## Abstract

The functional avidity is determined by exposing T-cell populations *in vitro* to different amounts of cognate antigen. T-cells with high functional avidity respond to low antigen doses. This *in vitro* measure is thought to correlate well with the *in vivo* effector capacity of T-cells. We here present the multifaceted factors determining and influencing the functional avidity of T-cells. We outline how changes in the functional avidity can occur over the course of an infection. This process, known as avidity maturation, can occur despite the fact that T-cells express a fixed TCR. Furthermore, examples are provided illustrating the importance of generating T-cell populations that exhibit a high functional avidity when responding to an infection or tumors. Furthermore, we discuss whether criteria based on which we evaluate an effective T-cell response to acute infections can also be applied to chronic infections such as HIV. Finally, we also focus on observations that high-avidity T-cells show higher signs of exhaustion and facilitate the emergence of virus escape variants. The review summarizes our current understanding of how this may occur as well as how T-cells of different functional avidity contribute to antiviral and anti-tumor immunity. Enhancing our knowledge in this field is relevant for tumor immunotherapy and vaccines design.

## 1. Introduction

CD8 T cells play a critical role in antiviral immunity, and a large number of studies in both human and mice indicate that antigen-specific CD8 T cells are directly involved in not only the control of viral replication, but also tumor growth [1–25]. Especially CD8 T-cell immunity against HIV replication, and thus the prevention of the disease progression, is well documented. This is primarily based on the following observations: (a) depleting CD8 T cells in the macaque model of AIDS leads to a loss of control of virus replication [4, 5, 26], (b) HIV-specific T-cell responses can be detected in previously virus exposed but presently uninfected individuals [27–30], and (c) a higher numbers of polyfunctional T cells are found in individuals with nonprogressive infection or in the so-called “elite controllers” [9–11], although there is a long-term debate as to whether this is a cause or a consequence of viral control [31]. Polyfunctional T cells characteristically show high IL-2 expression and strong ability to upregulate granzyme B and perforin. They have a high proliferative capacity and are superior in controlling HIV infection. Furthermore, it has been demonstrated by the whole genome analyses that HLA class I alleles are the genetic factors most strongly associated with nonprogressive infection [8, 15–22, 32, 33].

The aforementioned polyfunctionality is a well-established important indicator for the ability of T cells to control a virus infection. However, this parameter does not reflect the ability of how a T-cell or a population of T cells responds to a specific antigen. Instead, the polyfunctionality is usually assessed upon exposing T cells to peptide-MHC [pMHC] ligands at close to saturating concentration. In this situation, it can be that T cells show similar cytokine response patterns, although T cells might respond significantly different upon exposure to limited or physiologically relevant amounts of a ligand *in vivo* and *in vitro*.

In contrast, the functional avidity is a biological measure that describes how well a T-cell responds *in vitro* to a given concentration of a ligand. By definition, T cells with high functional avidity respond in *in vitro* tests to very low antigen doses, while T cells of lower functional avidity require higher amounts of antigen before they mount an immune response similar to that of high-avidity T cells. The functional avidity can be considered as a quantitative determinant of the activation threshold of a T-cell clone.* In vivo,* T cells of high and low avidity are exposed to similar antigen doses, but numerous correlations exist between the functional avidity and the effectiveness of an antiviral immune response, some of which will be discussed later in this paper. Of note, *ex vivo* studies have shown that distinct T-cell functions (e.g., proliferation, cytokines production, etc.) are triggered with different thresholds [34–37].

The purpose of this paper is to provide information on what distinguishes the functional avidity from other parameters used to describe the ability of T cells to recognize antigen and to summarize the known factors that determine the functional avidity of T cells as well as the functional avidity maturation of a T-cell population. The latter refers to increases of the overall functional avidity with which a polyclonal population responds to antigen. Moreover, we suggest that combining functional avidity assessment and polyfunctional analysis might lead to better predictions concerning the ability of a T-cell population to control a chronic infection than when both tests are independently performed. Finally, we will critically discuss the general consensus that high-avidity CD8 T-cell responses are always better in controlling virus infections by presenting evidence that this might not be the case in chronic infections, particularly during HIV infection.

## 2. Factors Impacting the Functional Avidity of a T-Cell Clone

The functional avidity inversely correlates with the antigen dose that is needed to trigger a T-cell response. It is determined by *ex vivo* quantification of biological functions such as IFN-*γ* production, cytotoxic activity (ability to lyse target cells), or proliferation. The concentration needed to induce a half-maximum response (EC_50_) is often used to describe the functional avidity of T cells. In particular, it can be used to describe how monoclonal but also antigen-specific polyclonal T-cell populations respond to antigen stimulation.

**Figure 1:**
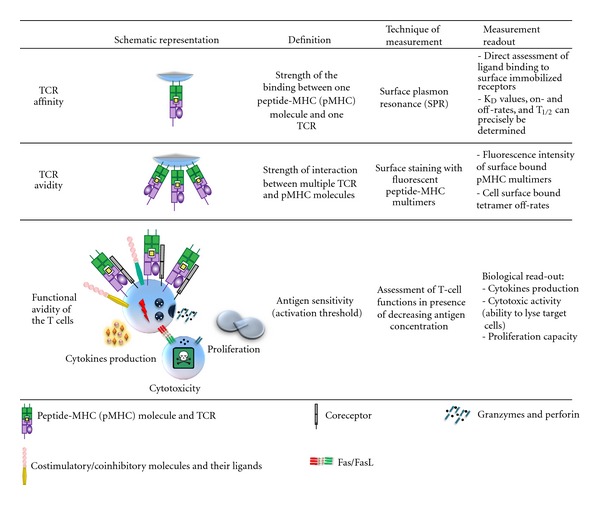
Schematic representation, definition, technique of measurement and readout of TCR affinity and functional avidity.

The functional avidity of a T-cell clone (Figure 1) is primarily impacted by (a) the affinity of the TCR for the pMHC-complex, that is, the strength of the interaction between the TCR and pMHC [38, 39], (b) expression levels of the TCR and the CD4 or CD8 coreceptors, and (c) the distribution and composition of signaling molecules [40, 41] as well as expression levels of molecules that attenuate T-cell function and TCR signaling.

### 2.1. Affinity, Avidity, and Functional Avidity of a T-Cell Clone

The terms affinity, avidity, and functional avidity are often incorrectly interchangeably used. The TCR affinity (Figure 1) refers to the physical strength of the monomeric interaction between the TCR and a pMHC-complex [42, 43]. The dissociation constant (K_D_) for different pMHC-TCR pairs have been determined by surface plasmon resonance [44]. Several reports indicate that a lower K_D_ and thus a stronger interaction lead to a better T-cell response [45, 46]. Another parameter that has been shown to influence the T-cell response efficacy is the half-life (*t*_1/2_) value of the interaction between the TCR and the pMHC-complex; longer *t*_1/2_ times also result in more potent T-cell stimulation [45, 47, 48]. It still remains controversial which of these two parameters, the K_D_ or the *t*_1/2_ value, offers a better prediction of how T cells respond to antigen stimulation [48]. Low K_D_ values result from slow off-rates and/or rapid on-rates. Thus, pMHC-TCR interactions, which have a long *t*_1/2_ time, usually also show a low K_D_ value. This relationship may in parts explain why both K_D_ and the *t*_1/2_ time have been reported to correlate with the efficiency of T-cell activation. However, both low K_D_ and long *t*_1/2_ values are thought to permit completion of intracellular signaling cascades leading to T-cell activation [42, 49].

However, a clean biochemical determination of K_D_ values and *t*_1/2_ times is rather complicated, and it requires the availability of soluble pMHC-complexes and a soluble form of the TCR. Moreover, it needs to be considered that the binding kinetics can significantly vary depending on whether the interaction is measured with soluble or membrane-bound ligands [50]. Alternatively, a more practical but less precise way to assess the strength of pMHC-TCR interaction is to stain living T cells with pMHC-multimers. Binding kinetics can then be determined by measuring fluorescent intensity of cell-surface bound multimers [51–53]. As the latter involves binding of a ligand via multiple interactions (i.e., the pMHC-multimers bind to more than one TCR), such measurements are best described by the term avidity, which is normally used to refer to the strength of multimeric receptor-ligand engagement (Figure 1).

In contrast to the physical parameters affinity and avidity, the functional avidity describes how well a T-cell responds to antigens. Though all of the three parameters correlate in most cases, that is, high-affinity T cells often have a high functional avidity, this does not need to be the case. There are several factors besides the antigen recognition ability of the TCR that can impact the T-cell response. In principle, T cells could express a high-affinity TCR, but due to other factors, for example, inhibitory molecules, it might show a very weak response to antigen stimulation. Thus, determining the functional avidity is not only often more practical, but is also the only one out of the three parameters that actually describes the functional outcome of the stimulation.

Notably, the level of TCR expression impacts the functional avidity. Even though T cells are functional when they express very little TCR, it has been shown that reduced expression levels go along with decreased ability to respond to antigen [54]. T cells with reduced TCR expression levels are impaired in their proliferative capacity and in their ability to secrete IL-2 and IFN-*γ* [55].

### 2.2. Coreceptor Expression Impacts the Functional Avidity of a T-Cell Clone

CD4 and CD8 coreceptors bind to the MHC classes II and I, respectively [56], and stabilize the pMHC-TCR interaction. This is for instance illustrated by the fact that pMHC-multimers bearing a mutation in the CD8 binding site bind less efficiently to T cells [57], and this is particularly prominent when a TCR binds with low-affinity to a pMHC-complex [58–60]. The importance of the coreceptor engagement for pMHC-binding to the TCR is also underlined by observations that antibodies against the CD8 coreceptor can decrease or block the extent to which pMHC-multimers bind to a TCR. There are even antibody clones enhancing the binding [61]. This might occur by inducing a conformation that facilitates better binding of the coreceptor to the MHC. This antibody enhancement or blockade is even more critical when a TCR engages a low-affinity ligand [61]. Moreover, coreceptor engagement supports TCR signal transduction by bringing Lck in close proximity to the TCR complex [62, 63]. Reducing this coreceptor engagement of Lck lowers the TCR sensitivity to antigen stimulation and thus decreases induction of effector function [39, 64]. However, the enhancement of the coreceptor MHC binding is more critical for low than high-affinity T-cell clones. Thus, high-affinity pMHC-TCR interactions are in contrast to low-affinity binding characterized by a relative CD8-independence for both T-cell proliferation and cytotoxicity as well as for multimer binding [57, 58, 60].

The aforementioned observations indicate how critically the presence or absence of the coreceptor impacts the ability of T cells to respond to a pMHC-complex. In addition, several examples indicate the extent to which variations in coreceptor expression levels or binding ability to MHC molecules impact T-cell function. In mice, downregulation of CD8 expression and reduced ability of T cells to respond to antigen have been seen following* Listeria monocytogenes* or *Vaccinia virus *infection [65]. Moreover, there is a polymorphism in the *α*3 domain of the HLA-A*68 molecule resulting in weak binding of the CD8 coreceptor to the MHC. When the HLA-A*68 sequence is artificially altered to restore CD8 binding, then this altered molecule is recognized by TCRs that fail to respond to normal HLA-A*68. Thus, restoring coreceptor engagement rescues cytokine production and T-cell proliferation, even though the pMHC-TCR interaction itself remains unchanged [66, 67]. Moreover, self-antigen-specific T cells can downregulate CD8 expression to reduce their functional avidity and thereby their autoreactive potential [68].

### 2.3. Alterations in TCR Signaling Can Impact the Functional Avidity

TCR signaling is initiated when Lck (Src family tyrosine kinase) phosphorylates immunoreceptor tyrosine-based activation motifs (ITAM) within the CD3*ζ* molecule. This provides a docking site for ZAP-70, which in turn gets phosphorylated by Lck [69]. Activated ZAP-70 leads to the recruitment and phosphorylation of linker for activation of T-cell (LAT) and SH2-domain-containing leukocyte protein of 76 kDa (SLP-76). This initiates a signaling cascade that leads to Ca^2+^ mobilization as well as to the activation of the mitogen-activated protein kinase (MAPK) signaling pathway [70, 71].

Different lines of evidence indicate that T cells can adjust or tune the sensitivity of their signaling apparatus indicating that functional avidity is not fixed [72]. In the thymus, TCR-signaling sensitivity is thought to be augmented by a micro RNA (miR181a), and this occurs via targeting multiple phosphatases that otherwise inhibit the TCR signal [73, 74]. This goes along with observations that T cells respond to ligands in the thymus to which they much less effectively respond in the periphery [75, 76]. Furthermore, TCR signal transduction is thought to be fine tuned by inhibitory molecules such as CD5 [77]. CD5 expression levels presumably correlate with the strength with which a T-cell clone responded to its positive selecting ligand [77]. CD5 is an immune-tyrosine-based inhibition motif-bearing receptor that could antagonize overt TCR activation in peripheral T cells [78] and has been shown to be involved in peripheral tolerance by adjusting T-cell reactivity [79]. Along those lines, higher antigen sensitivity is determined by the superior ability of high-avidity T cells to achieve threshold levels of CD3*ζ* phosphorylation through increasing the amount of activated Lck [80, 81].

If and how these and other pathways impact the functional avidity of T cells need to be better determined. However, the ability of peripheral T cells to alter their antigen reactivity has been reported. Deprivation from MHC molecules has been shown to increase CD4 T-cell reactivity [82], but also the opposite effect has been observed [83].

## 3. Factors Impacting the Functional Avidity at the Population Level

The overall functional avidity of a heterogeneous oligoclonal T-cell population that forms during an infection [84–88] is primarily impacted by the ratio of recruited clones with high *versus* low functional avidity. Thus, the stimulatory potency and the range of functional avidity that antigen-presenting cells [APC] recruit during an immune response strongly impact the avidity of the emerging T-cell population. Whether or not an APC is able to recruit not only T cells with high but also low functional avidity is critically impacted by the net level of costimulatory and inhibitory molecules, but also by the magnitude of antigen presentation.

### 3.1. Impact of Costimulatory and Inhibitory Molecules

The interaction between T cells and DC involves several molecular contacts between not only costimulatory but also inhibitory molecules. Expression levels of these molecules can modulate the T-cell activation threshold which in turn impacts the functional avidity of the emerging T-cell population; that is, if the threshold is for a particular reason very high, then only T-cell clones which have a rather high functional avidity will be activated and the overall functional avidity of the merging population will be high.

An example for the modulation of T-cell activation thresholds is the CD70/CD27 mediated costimulation which enhances the response to low-affinity ligands [89]. Moreover, it has been shown that APCs which express higher levels of B7.1, but also ICAM and LFA3, induce T cells with higher functional avidity. Those were shown to proliferate more vigorously, produce more cytokines, and kill target cells more efficiently in both primary and secondary T-cell responses [90, 91]. Similar observations were made using a combination of B7 costimulations and *α*-CTLA-4 anti-body-mediated blockade [92].

On the other hand, APCs can express molecules that induce negative signals in CD8 T cells such as PD-L1. The expression of such inhibitory receptors is mostly driven by persisting antigen stimulation in chronic infections or tumors [93, 94].

### 3.2. Antigen Doses and Antigen Presentation

Several *in vitro* and *in vivo* studies indicate that antigen exposure influences the antigen sensitivity of the emerging T-cell population [95–97]. It was shown that CD8 T cells expanded by low doses of peptide successfully lyse target cells expressing less antigen and mediated increased viral clearance than CD8 T cells stimulated with high peptide doses [97]. DC presenting different densities of pMHC-complexes had distinct influence on the functional avidity of responding CD8 T cells in immunized mice. In particular, low antigen doses were associated with high avidity and higher capacity of recall responses to recognize melanoma cells [96]. Thus, the ligand density that is presented during an infection and during T-cell priming can impact what types of T cells emerge [40], which seems to be particularly important in the context of vaccination [97].

The amount of pMHC-complexes presented by an APC critically depends on the stability of the individual pMHC-complex. Surface pMHC-turnover rates also impact which types of T-cell clones become activated [98, 99]. Interestingly, DCs seem to be able to present pMHC-complexes much longer than other cells, which likely supports their nonredundant role in initiating T-cell responses [100].

More recently, the impact of the peptide dose on CD8 T-cell avidity has been investigated in melanoma patients vaccinated with different doses of Melan-A/MART-1 peptide. Melan-A-specific CD8 T cells from patients vaccinated with low peptide doses had functionally high-avidity T cells with low CD8 dependency. In particular, they showed enhanced degranulation and cytotoxic activities and lower levels of CD8 expression [101]. These observations facilitated the development of new immunotherapy approaches against cancer and chronic infections.

## 4. Functional Avidity Maturation

The functional avidity of a T-cell population often increases during the course of an immune response and following pathogen reexposure [102]. Along with that, enhancement in pMHC-multimer binding has been reported [103]. Two principle mechanisms have been shown to contribute to the avidity maturation phenomenon. Clonal remodeling in the population of antigen-specific T cells occurs massively in primary infections [104], recall responses [105, 106], and for instance during persisting infection like CMV in humans [107]. During this remodeling, the progeny of T-cell clones with high functional avidity become more prevalent. In primary infections, the differences in expansion length between T-cell clones with high or low functional avidity account for this phenomenon [104]. However, the mechanisms driving clonal remodeling in secondary infections are less clear, but it is likely caused by antigen competition between high and low-affinity T cells clones [108] and possibly by alterations in the T-cell stimulation threshold.

Moreover, several lines of evidence suggest that even T cells expressing the same TCR can differ in their functional avidity and that the latter depends on the state of differentiation of T cells. For instance, it has been shown that during an LCMV infection, the functional avidity of TCR transgenic T cells increases [81]. Moreover, it has been observed that memory T cells can exhibit a higher functional avidity than that of naïve T cells [109, 110]. Several mechanisms have been proposed to contribute to the maturation of the functional T-cell avidity at the clonal T-cell level. Those include (1) the formation of clusters that comprise several TCRs and other molecules able to reinforce the immunological synapses and changes in the cholesterol content of the membrane contribute to such differences [111–113] and (2) the optimization of the signal transduction machinery, for example, by increasing the amount and the basal phosphorylation levels of signaling molecules [81, 114]. Moreover, it has been shown that the expression of Lck correlates with the production of IFN-*γ*, whereby minor increases in Lck expression lead to major increases in IFN-*γ* production [57]. In contrast, it has also been reported that the functional avidity can decrease [65] or remain similar (as seen for OT-1 TCR transgenic T cells [104]) during an infection, and whether or not memory T cells are truly more sensitive than naïve T cells remains controversial.

Overall, functional avidity maturation allows faster virus clearance/control at the time of antigen reencounter and a progressive acquisition of coreceptor binding and costimulatory signal independency [81, 115, 116]. In the context of peripheral tolerance, however, the continuous exposure to antigen in the context of molecular mimicry might lead to affinity maturation which in turn may result in autoimmunity [72]. Besides this consideration, it has also been shown that autoreactive T cells undergo limited avidity maturation [117].

In contrast to these processes that follow acute infection, the dynamics in the T-cell population in chronic infections appear to be different. We recently demonstrated that HIV-specific CD8 T cells undergo a massive TCR renewal for instance following a virus rebound [84]. Interestingly, it has been observed that changes in TRBV populations overtime go along with a loss of low-avidity T cells clones or more generally speaking an increase in functional avidity [118]. These observations will be discussed in more details in the final sections of this paper.

## 5. The Functional Avidity of T Cells as a Correlate of Immune Protection

There is a general consensus that higher functional avidity CD8 T-cell responses are of higher efficacy to eliminate cancer cells and to clear acute virus infections, a notion that is supported by a large number of reports [103, 106, 119–121].

It was for instance shown that high functional avidity Tax-specific CD8 T-cell lines—which use a very diverse TCR repertoire—are superior in their ability to eliminate HTLV-1-infected cells than low-avidity Tax-specific CD8 T cells. These cells were also able to recognize a latent Tax level (detectable only by RT-PCR) produced by adult T-cell leukemia cells (ATLs), thus possibly leading to the prevention of HTLV-1 infection [122].

For tumors, high functional avidity T cells mediate better T-cell responses [119], though it needs to be said that tolerance-enforcing mechanisms effectively remove high-avidity self- and tumor-antigen reactive T cells [123, 124]. Thus, tumor-reactive T cells will in most cases have a lower functional avidity than what can be observed during acute infections.

Low antigen expression and absence of inflammatory and costimulatory signals may be partially responsible for the low immunogenicity of many tumor cells. The presence of higher-avidity CD8 T cells may be particularly relevant to overcome the tolerance to tumor antigens. This may be achieved by defining a combination of adjuvants and by regulating antigen doses in vaccines [97, 125, 126]. Indeed, higher-avidity T cells are preferentially triggered leading to a more rapid and effective target-cell elimination [14, 26, 97, 119, 127–129]. Consistently, in both humans and mice models, induction of higher-avidity CD8 T-cell responses promoted more efficient tumor rejection [101, 130] and earlier target cell lysis in the context of viral infection, reducing viral burden more effectively than low-avidity CD8 T cells. In addition, this activity does not depend on the frequency of antigen-specific CD8 T cells [131].

 There are also several studies correlating the presence of certain MHC alleles with differences in the diversity and functional avidity of T-cell clones that emerge during tumors or chronic infections. For example, H-2K^bm8^ mice express an H-2K allele that differs in four amino acids from H-2K^b^. Compared to C57BL/6 mice, the H-2K^bm8^ mice generate a different repertoire of high-avidity herpes simplex virus- (HSV-) specific T cells which correlates with a higher resistance to HSV infection [14].

Similarly, C57BL/6 mice are less susceptible to *respiratory syncytial virus* (RSV) infection than BALB/c mice. Among the many possible explanations, it has been observed that the H-2d alleles induce the generation of immunodominant antigen-specific CD8 T-cell populations that use a more restricted TCR repertoire, and that is less efficient in lysing target cells than what can be observed in C57BL/6 mice. This results in continuous T-cell stimulation and thus in cytokine-mediated immunopathology [132].

Furthermore, the strongest genetic association of the ability to effectively control HIV infections points at the MHC locus [32] and at the presence of certain MHC molecules such as HLA-B*57 [133]. In line with the earlier mentioned, the latter is thought to strongly impact the repertoire and the quality of HIV-specific T cells and thus enabling an enhanced virus control.

In contrast to these observations, the relevance of high- and low-avidity T cells in chronic virus infections and established tumors [86, 134, 135] remains to be determined, in particular, since some studies have challenged the superiority of high-avidity CD8 T cells [136, 137]. Indeed, low-avidity T cells (1) might better distinguish between tumors overexpressing self-antigens and healthy self-tissue [136] and (2) might during chronic viral infections and tumors be less sensitive to activation-induced cell death (AICD) [129, 138], senescence and exhaustion, leading to protracted survival of functionally-competent T cells, and (3) are less likely to induce viral or tumor escape [26, 134, 139].

In the context of chronic-controlled infections, such as CMV and EBV, the T-cell responses to immunodominant antigens have a more diverse repertoire with higher TCR avidity than that of subdominant clonotypes. However, they are also more prone to senescence [140]. In addition, *in vitro* stimulation at high antigen concentrations induces higher AICD [138] and more pronounced inhibition of proliferation in high-avidity than in low-avidity T cells [129]. Finally, we [36] and others [141] have shown that high-avidity CD8 T cells express higher levels of the T-cell exhaustion marker PD-1 than those of low-avidity CD8 T cells.

In addition, in the context of tumor immunity, skin depigmentation is considered as a good prognosis indicator in melanoma patients, since it is a sign of immune activation against tumor/self-antigens, and a high frequency of CD8 T cells was observed in depigmentated tissue from patients [137]. These were MC1R HLA-A2-specific, and despite harboring low functional avidity, they were cytolytic and produced IFN-*γ* and granzyme B [137]. Interestingly, immunization of mice tolerant to the hemagglutinin (HA) antigen and bearing a renal carcinoma overexpressing HA led to the expansion of low-avidity HA-specific CD8 T cells. These could only target tumor cells expressing high antigen doses, thus allowing the destruction of HA-over-expressing tumors but not healthy pancreatic cells [136].

However, it is worth mentioning that depending on the biology of the pathogen, T cells endowed with different functions and tropism are required, and, thus, a generalization of the features of a universally efficient T-cell response is complicated or may be not possible.

## 6. Functional Avidity as a Correlate of Control in HIV Infection

With regard to HIV infection, contrasting conclusions on the relationship between functional avidity and virus control have been reported [142–146]. Some studies indicated that protective HIV-specific CD8 T-cell responses (e.g., those observed in HIV-infected patients with nonprogressive infection) were of high functional avidity and mediated superior variants recognition [86, 135, 143, 147, 148]. In these studies, high-avidity CD8 T cells were not only mostly polyfunctional, endowed with potent virus suppressive activity, increased cross-reactivity, and associated with low levels of virus replication [86, 135, 147], but were also characterized by an increased T-cell turnover and senescence [86].

However, most of these studies focused on HIV-specific CD8 T-cell responses directed against only one epitope, and analyses were performed on single clones derived from T-cell expansion which may not reflect the *in vivo*/*ex vivo* profile of T cells [38, 39]. In addition, since the majority of studies reporting correlations between functional avidity and virus control are cross-sectional and not prospective studies in unselected populations, it is not possible to determine causality between avidity and virus control.

More recently, it was shown that gag-specific and HLA-B-restricted CD8 T-cell responses, usually associated with virus control [16, 149], have higher functional avidity than nef-, pol-, and env-specific CD8 T-cell responses [147]. Also, both gag-specific and HLA-B-restricted CD8 T-cell responses were of higher functional avidity in controllers than in noncontrollers. Finally, consistently with the above-mentioned studies, protective T-cell responses against KK10 and KF11 (restricted by HLA-B*2705 and B*5701, resp.) had higher functional avidity than all the other HLA-B-restricted epitopes [147].

Conversely, other studies indicated that the functional avidity of HIV-specific CD8 T cells is not different between patients with progressive or nonprogressive chronic infection or between gag- and other HIV-specific CD8 T cells [150] and also that uncontrolled virus replication seen in progressive HIV infection occurs despite the presence of high-avidity HIV-specific CD8 T-cell responses [142–146, 151, 152]. In this regard, we previously showed that polyfunctional virus-specific CD8 T-cell responses in the context of chronic viral infections were predominantly of low functional avidity [36]. In addition, when the avidity of two different CD8 T-cell clonotypes recognizing one HLA-B*35-restricted Pol epitope was analyzed, a 3fold difference in *t*_1/2_ for HLA-multimer interaction was found. In contrast to the clone of lower affinity, the one with higher affinity did not show cytotoxic activity, cytokine production, or proliferative capacity following stimulation with the cognate antigen [153]. Furthermore, CD8 T cells transduced with a high-affinity TCR showed greater binding activity toward the specific multimer, but impaired cytotoxicity [153].

Finally, it was also reported that high functional avidity T-cell responses preferentially led to viral escape, T-cell clonal exhaustion, and senescence [26, 86, 134, 139, 141, 154]. Indeed, CD8 T cells have distinct ability to select for escape mutations for the same epitope depending on HLA restriction. The HLA restriction which confers higher avidity for the epitope induces a substantially higher level of sequence variation and clonal turnover which in turn leads to faster viral escape [134] and T-cell senescence [86]. However, to some extent, the emergence of viral variants escaping recognition from higher avidity T-cell responses may also be interpreted as an argument to support the efficacy of high-avidity T cells against HIV. Furthermore, the specificity of CD8 T-cell responses is critical, since cells directed toward highly variable regions may nonetheless not be able to mediate virus control. In case, they might only cause the emergence of virus escape variants. Moreover, TCR avidity correlates with PD-1 expression levels, and, consistently, high-avidity CD8 T cells displayed an impaired survival in *in vitro* culture at low levels of antigen stimulation. *In vivo*, subdominant clonotypes not only are of low functional avidity and express lower PD-1 levels than those of dominant clonotypes, but also respond more efficiently to variant epitopes, thus displaying a greater capacity of cross-recognition [141]. Although increased PD-1 expression might also be interpreted as a marker of increased activation of higher avidity T cells [155], in the context of chronic infection such as HIV, PD-1 is predominantly considered as a marker of exhausted cells [156].

Although large-scale longitudinal studies are needed to further elucidate the dynamic relationships between functional avidity, immunodominance, and viral escape, the aforementioned information suggest that lower functional avidity T-cell responses might be more suitable in the context of HIV infection.

## 7. Functional Avidity of T Cells in Acute HIV Infection

A better understanding of the immune response during primary HIV infection (PHI) is of particular relevance, since HIV-specific CD8 T cells in PHI are temporally associated with the initial control of virus replication [2].

Of interest, Lichterfeld and colleagues suggested that high-avidity HIV-specific CD8 T-cell responses are present during early infection (defined as HIV seroconversion within 6 months) but are then selectively depleted overtime [118]. To our knowledge, there is no previous study addressing the issue of the functional avidity of HIV-specific CD8-T cells in a cohort of HIV-infected patients with very early acute infection (based on stringent criteria of enrollment).

**Figure 2:**
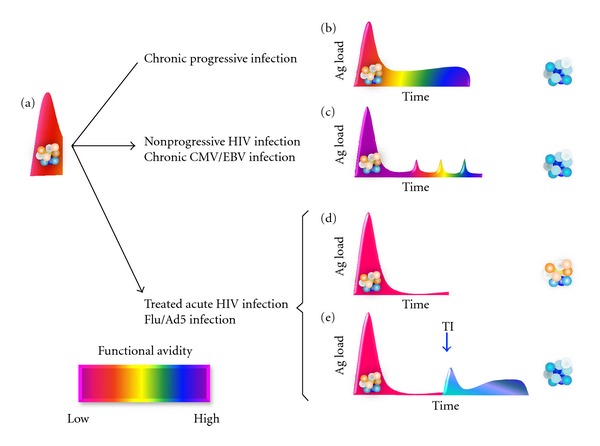
Proposed Model of the relationships between antigen exposure and functional avidity of T cells. Functional avidity of virus-specific CD8 T cells during (a) acute infection and then translation after transition to the chronic phase for (b) uncontrolled virus infection (such as progressive HIV infection) or (c) controlled but persistent virus infection (such as nonprogressive HIV, cytomegalovirus [CMV], or Epstein-Barr virus [EBV], or (d) after virus clearance (such as influenza [Flu] or adenovirus [Ad5], or early treatment of acute HIV infection). (e) Increase in the functional avidity of HIV-specific CD8 T cells of patients treated during acute infection who interrupted the antiretroviral therapy [TI] and experienced a virus rebound.

However, we recently had the opportunity to investigate the functional avidity of HIV-specific CD8 T-cell responses in a *true* PHI cohort (i.e., presence of an acute clinical syndrome, a negative HIV antibody test, a positive test for HIV RNA in plasma, and presence of fewer than three positive bands in a Western blot) [157]. In this context, we observed that the functional avidity of HIV-specific CD8 T-cell responses was significantly lower in PHI than in chronic infection and remained low after several years of antiretroviral therapy (Vigano and Harari, unpublished observation). Conversely, we noted (Figure 2) an increase in the functional avidity of HIV-specific CD8 T cells in patients experiencing a virus rebound following treatment interruption [84].

These observations might be explained by two nonmutually exclusive mechanisms: first, the progressive selection of clones with higher functional avidity and, on the other hand, the recruitment of new clones with higher functional avidity. The potential combination of these mechanisms would induce a modification of the TCR repertoire [84, 103, 106, 107, 118].

## 8. Perspectives and Hypothesis

Detailed monitoring of phenotypes and functional characteristics of T cells in different viral infection has strongly augmented our understanding of the relationship between viral infections and the immune response they induce. We recently made thorough comparisons of the types of T cells responding to infections that the immune system rapidly clears (*Influenza* (Flu) or *Adenovirus* (Ad5)), or infections caused by CMV and EBV as well as HIV infections in the acute and chronic phase. We saw that the functional avidity of T cells specific to Flu and Ad5 was similar to that of T cells in the acute phase of HIV infection. In contrast, significantly higher functional avidities of T cells were noticed in the chronic progressive and nonprogressive HIV infection, but interestingly those were comparable to the functional avidity seen during chronic CMV and EBV infections. Furthermore, when patients were treated during acute infection but experienced a virus rebound following treatment interruption (TI), the functional avidity of HIV-specific CD8 T cells increased (AH and SV unpublished observations).

## 9. Summary

Functional T-cell avidity is a critical attribute of antiviral and antitumoral immunity. The strength of interaction between the TCR and pMHC-molecule, expression levels of the coreceptors, as well as signaling particularities are pivotal in determining the functional avidity of a T-cell clone.

It is well established that T-cell populations can over the course of infection or upon multiple exposure to infections or infectious exacerbation undergo significant changes in the ability to recognize cognate antigen. The later is at a first glance somewhat surprising as T cells, unlike B cells, express a fixed TCR and cannot undergo somatic hypermutation. Predominantly, the clonal composition impacts the functional avidity of the T-cell population and avidity maturation. In addition to the reinforcement of the immunological synapses through the formation of TCR clusters, the optimization of the signal transduction machinery further contributes to avidity maturation. However, the understanding of mechanisms underlying this phenomenon is still limited, and further studies need to be undertaken to better understand how all these possible variations and likely many yet unknown ones impact the functional avidity of T cells when responding to different types of infections.

In the context of viral infections, functional avidity maturation allows faster virus clearance by recall T-cell responses. However, the role of high versus low functional avidity T cells in chronic viral infections such as HIV remains unclear. Here, it needs to be considered that high-avidity T cells exhibit greater T-cell exhaustion and lead to rapid emergence of escape variants suggesting a pivotal role of low-avidity T cells.

Studies to better delineate the factors influencing the functional avidity of T-cell responses are relevant in order to allow fine tuning of the profile of vaccine-induced T cells. We consider that the goal of vaccination or immunotherapy against acute infections and to induce pathogen clearance should be the induction of high-avidity T cells, since such cells most effectively eliminate infected cells. Conversely, when pathogen clearance cannot be achieved, then the ultimate goal is to provide durable control of a persistent pathogen. A vaccine that deals with such a situation should be designed to generate low-avidity T cells, since those might be more suitable to generate a pool of long-lasting effector T cells in a situation of chronic infection.
